# Pterostilbene Exerts Antitumor Activity via the Notch1 Signaling Pathway in Human Lung Adenocarcinoma Cells

**DOI:** 10.1371/journal.pone.0062652

**Published:** 2013-05-03

**Authors:** Yang Yang, Xiaolong Yan, Weixun Duan, Juanjuan Yan, Wei Yi, Zhenxin Liang, Ning Wang, Yue Li, Wensheng Chen, Shiqiang Yu, Zhenxiao Jin, Dinghua Yi

**Affiliations:** 1 Department of Cardiovascular Surgery, Xijing Hospital, The Fourth Military Medical University, Xi’an City, China; 2 Department of Thoracic Surgery, Tangdu Hospital, The Fourth Military Medical University, Xi’an City, China; 3 Department of Prosthodontics, School of Stomatology, The Fourth Military Medical University, Xi’an City, China; Cincinnati Children's Hospital Medical Center, United States of America

## Abstract

Although pterostilbene (PTE) has been shown to have potent antitumor activities against various cancer types, the molecular mechanisms of these activities remain unclear. In this study, we investigated the antitumor activity of PTE against human lung adenocarcinoma in vitro and in vivo and explored the role of the Notch1 signaling pathway in this process. PTE treatment resulted in a dose- and time-dependent decrease in the viability of A549 cells. Additionally, PTE exhibited strong antitumor activity, as evidenced not only by a reduced mitochondrial membrane potential (MMP) and a decreased intracellular glutathione content but also by increases in the apoptotic index and the level of reactive oxygen species (ROS). Furthermore, PTE treatment induced the activation of the Notch1 Intracellular Domain (NICD) protein and activated Hes1. DAPT (a gamma secretase inhibitor) and Notch1 siRNA prevented the induction of NICD and Hes1 activation by PTE treatment and sensitized the cells to PTE treatment. The down-regulation of Notch signaling also prevented the activation of pro-survival pathways (most notably the PI3K/Akt pathway) after PTE treatment. In summary, lung adenocarcinoma cells may enhance Notch1 activation as a protective mechanism in response to PTE treatment. Combining a gamma secretase inhibitor with PTE treatment may represent a novel approach for treating lung adenocarcinoma by inhibiting the survival pathways of cancer cells.

## Introduction

Lung cancer is the leading cause of cancer-related death worldwide. Non-small cell lung cancer (NSCLC) subtypes (adenocarcinoma, squamous cell carcinoma and large cell carcinoma) account for 80–85% of all lung cancers. The majority of patients diagnosed with NSCLC are diagnosed with advanced stages and have inoperable local or distant metastases [1]. Although there have been significant advances in the treatment of lung adenocarcinoma due to the introduction of novel chemotherapies combined with targeted agents, the overall survival rate remains low. Cancers eventually develop resistance to standard treatments through the activation of pro-survival pathways in tumors [2]. In addition, these treatment regimens often have obvious side effects for patients and are inadequate for treating the disease. The magnitude of this problem indicates that there is a great need for novel therapeutic agents, specifically chemopreventive agents derived from less harmful natural materials.

Pterostilbene (3,5-dimethoxy-4′-hydroxystilbene, 4′-[(1E)-2-(3,5-dimethoxyphenyl) ethenyl]phenol, PTE), a natural dimethylated analog of resveratrol from blueberries, is known to have diverse pharmacological activities, including anticancer, anti-inflammation, antioxidant, anti-proliferative and analgesic properties [3]. The dietary administration of high doses of PTE is not toxic to mice [4]. PTE has potent antitumor activities with low toxicity in various cancer types, including breast cancer [5], liver cancer [6] and prostate cancer [7]. Under most circumstances, PTE is either equally or significantly more potent than resveratrol. PTE may have greater biological activity due better bioavailability resulting from the substitution of a hydroxyl group with a methoxyl group, which increases the molecule’s lipophilicity [8]. Studies have also shown that resveratrol can induce the apoptosis of various types of cancer cells through the regulation of the Notch signaling pathway [9], [10]. However, the effects of PTE on human lung adenocarcinoma and the mechanisms responsible for these effects have not been elucidated.

The Notch signaling pathway is a highly conserved signaling pathway that can affect cellular activities including proliferation, migration, growth, differentiation and death. To date, a single Notch receptor (Notch1-4) and two types of Notch ligands (Jagged1/2 and Delta1/3/4) have been discovered in mammals. The genes downstream of Notch in the signaling pathway include Hairy and enhancer of split 1 (Hes1) and the Hairy-related transcription (HRT) factor family [11]. The activation of Notch signaling can induce the expression of multiple targets involved in cellular proliferation, such as Cyclin D1 and survivin [12].

The activation of the Notch1 pathway is increasingly being studied as a novel mechanism for tumorigenesis [9]–[11]. Notch1 was originally found to be overexpressed in T-cell leukemias as the result of an oncogenic translocation, and since then, the Notch1 pathway has been shown to be activated in multiple tumor types, including lung adenocarcinomas [13]. Further contributing to oncogenesis, the activation of the Notch1 pathway induces pro-survival signals that are associated with resistance to chemotherapy. The overexpression of Notch1 increases the resistance of lung cancers to cisplatin and paclitaxel [14], the resistance of breast cancers to melphalan and mitoxantrone [15] and the resistance of cervical cancers to doxorubicin [16]. Notch1 signaling can also contribute to the survival of cancer cells by protecting cells from apoptosis because this signaling pathway activates targets involved in cellular survival, such as phosphoinositide kinase-3 (PI3K)/Akt [17], survivin [18] and Bcl-X_L_ [19]. However, the relationship between Notch1 activation and the sensitivity of tumor cells to cytotoxic agents in lung adenocarcinoma has not been examined. In this study, we investigated the antitumor activities of PTE against human lung adenocarcinoma in vitro and in vivo and the role of the Notch1 signaling pathway in these activities.

## Materials and Methods

### Materials

DAPT (a gamma secretase inhibitor), MTT [3-(4,5-dimethylthiazol-2-yl)-2, 5-diphenyltetrazolium bromide], LY294002 (LY, a specific inhibitor of PI3K/Akt signaling), 3,3′-diaminobenzidine (DAPI), dithiothreitol (DTT) and 2′,7′-dichlorofluorescein diacetate (DCFH-DA) were purchased from the Sigma-Aldrich Company (St. Louis, MO, USA). The glutathione (GSH) kit was obtained from Cayman Chemical (Ann Arbor, MI, USA). PTE, Notch1 siRNA and the antibodies against NICD, Hes1, Cyclin D1, survivin, Bax and cytochrome c were obtained from Santa Cruz Biotechnology (Santa Cruz, CA, USA). Akt siRNA and the antibodies against Nicastrin, Presenilin-1, p-Akt^ser473^, Akt, p-S6^Ser235/236^, S6, mammalian target of rapamycin (mTOR), p-mTOR^ser2448^, DNA-dependent protein kinase (DNA-PK) and GAPDH were obtained from Cell Signaling Technology (Beverly, MA, USA). Terminal deoxynucleotidyl transferase dUTP nick-end labeling (TUNEL) kits were purchased from Roche (Mannheim, Germany). The fluorescent dye JC-1 was purchased from the Beyotime Institute of Biotechnology (Nanjing, Jiangsu, China). The rabbit anti-goat, goat anti-rabbit and goat anti-mouse secondary antibodies were purchased from the Zhongshan Company (Beijing, China).

### Cell Culture and Treatment

Human A549 lung adenocarcinoma cells were obtained from the Cell Culture Center, Chinese Academy of Medical Sciences (Shanghai, China). The cells were grown in Dulbecco's modified Eagle's medium (Gibco, Grand Island, NY, USA) supplemented with 10% fetal bovine serum (Gibco), L-glutamine (2 mM), penicillin (100 units/ml), streptomycin (100 units/ml) and HEPES (25 mM). The cells were maintained in the presence of 5% CO_2_ at 37°C. A PTE stock solution was prepared in DMSO and diluted with culture medium immediately prior to the experiment. DMSO (0.01%) was used as the control. First, the cells were treated with PTE (1.5, 3 or 6 µM). Then, the cells were treated with PTE (6 µM) in the absence or presence of DAPT (a known Notch inhibitor, 10 µM), Notch1 siRNA and Akt siRNA for different lengths of time. After the treatments were performed, the cells were harvested for further analysis.

### Analysis of Cell Viability

An MTT assay was performed to assess the viability of the lung adenocarcinoma cells. After the cells were treated and washed with PBS, 100 µL of 0.5 mg/mL MTT solution in phenol red-free DMEM was added to the cells, and the samples were incubated for 4 h at 37°C. Finally, 100 µL of N,N-dimethylformamide was added to each well, and the samples were shaken for 15 min at 37°C. The results were analyzed at 490 nm using a microplate reader (SpectraMax 190, Molecular Device, USA), and the cell viability was expressed in terms of the optical density (OD). In addition, the cell morphology was observed under an inverted/phase contrast microscope, and images were taken with an Olympus BX61 microscope (Japan).

### Analysis of Cell Apoptosis

The level of cellular apoptosis was analyzed by performing a TUNEL assay using an in situ cell death detection kit. A double-staining technique was used according to the manufacturer’s instructions. After the lung adenocarcinoma cells were fixed in paraformaldehyde (4%) for 20 min, the TUNEL assay was performed to stain the nuclei of the apoptotic cells (green), and DAPI was used to stain the nuclei of all cells (blue). The apoptotic index was expressed as the percentage of green cells.

### Analysis of the Cell Mitochondrial Membrane Potential (MMP)

The MMP was estimated by flow cytometry after staining with the fluorescent dye JC-1. When a cell is in a normal state, the MMP is high and JC-1 predominantly exhibits red fluorescence. When a cell is in an apoptotic or necrotic state, the MMP is reduced and JC-1 is present as a monomer; in this for, the dye emits green fluorescence. A change in the florescence from red to green indicates a decrease in the MMP. Cells in 6-well plates were treated with PTE (1.5, 3 or 6 µM) for 24 h, washed with PBS and incubated with the JC-1 working solution for 20 min at 37°C in the dark. The cells were washed with PBS and resuspended in 500 µl of PBS. The stained cells were analyzed using a FACScan flow cytometer equipped with the FACStation data management system running Cell Quest software (all from Becton Dickinson, San Jose, CA). The results were expressed as the proportion of cells with a low MMP.

### Analysis of Intracellular ROS Generation

DCFH-DA passes through cell membranes and is cleaved by esterases to yield DCFH. ROS oxidize DCFH, generating the fluorescent compound dichlorofluorescein, which can be used for quantification. After being treated with PTE (1.5, 3 or 6 µM) for 24 h, the cells were trypsinized and subsequently incubated with DCFH-DA (20 µM) in PBS at 37°C for 2 h. After incubation, the DCFH fluorescence of the cells in each well was measured using an FLX 800 microplate fluorescence reader with 530 nm as the emission wavelength and 485 nm as the excitation wavelength (Biotech Instruments Inc., USA). The background was determined using cell-free conditions. The fluorescence intensity in the control group was defined as 100%.

### Analysis of the Intracellular GSH Levels

The GSH and oxidized glutathione (GSSG) levels were determined in cells using a glutathione kit as described previously [20]. Briefly, cells were plated at a density of 1×10^6^ in 100 mm culture dishes and allowed to attach overnight. The cells were treated on the second day with PTE. The cells were collected by scraping and washed with PBS, and the cell lysate was used to determine the GSH level (using the above-mentioned kit) according to the manufacturer's instructions. To determine the GSSG levels, GSH was masked using 2-vinyl pyridine for 1 h before the assay. The samples were read at 405 nm at 5 min intervals for 30 min. The GSH and GSSG levels were quantified by comparison with standards and were normalized to the total protein content. The results were expressed as the total GSH (% of control) or the GSH/GSSG ratio, and a reduced form of GSH or an oxidized form of GSH (GSSG) was used as the standard.

### Transfection of siRNA

The cells were plated onto 6-, 24- or 96-well plates and allowed to grow to sub-confluency. The cells were then transfected with Notch1 siRNA at 50 pmol/L or with Akt siRNA at 50 nM using Oligofectamine (Invitrogen, Carlsbad, CA) for 48 h. The cells were also transfected with a control siRNA with a random sequence (Santa Cruz) at 50 pmol/L. Subsequently, the cells were prepared for further experiments.

### Immunofluorescence Assay

After the cells were fixed in paraformaldehyde (4%) for 20 min, they were permeabilized in 0.1% Triton X-100 for 10 min and blocked in 5% bovine serum albumin for 30 min at room temperature. Next, the cells were incubated with anti-Notch1 and anti-Hes1 goat polyclonal antibodies (1∶200) overnight at 4°C. Following washes with PBS, the cells were incubated with a rabbit anti-goat secondary antibody conjugated to TRITC or FITC (1∶200) for 2 h. Subsequently, the cells were incubated with DAPI (0.02 mg/ml) for 2 min, washed with PBS and wet-mounted using glycerol (50% v/v). The images were taken using a fluorescence microscope (BX51, Olympus, Japan) equipped with a CCD camera (DP70, Olympus, Japan).

### Antitumor Activity in a Xenograft Model

Male athymic nude mice were purchased from Laboratory Animal Centre of the Fourth Military Medical University. The mice were housed and maintained under specific pathogen-free conditions in facilities approved by the American Association for Accreditation of Laboratory Animal Care and in accordance with current regulations and standards of the United States Department of Agriculture, United States Department of Health and Human Services. The present study was performed according to the Guide for the Care and Use of Laboratory Animals published by the US National Institutes of Health (National Institutes of Health Publication No. 85-23, revised 1996) and approved by the Ethics Committee of the Fourth Military Medical University. All surgery was performed under sodium pentobarbital anesthesia, and all efforts were made to minimize suffering. A549 cell tumor xenografts were established by subcutaneously injecting 1×10^6^ cells into the right flanks of 4- to 6-week-old male athymic nude mice. Based on the data from a pilot study, we initiated treatment when the tumor volume reached approximately 100 mm^3^. The tumor volumes (V) were calculated using the following formula: V = A×B^2^/2 (A = largest diameter; B = smallest diameter). The mice were randomly divided into six groups (n = 5): mice treated with PBS only (control group), mice treated with PTE (100 mg/kg) in PBS (PTE group), mice treated with PTE (100 mg/kg) and DAPT (10 mg/kg) in PBS (PTE+DAPT group), mice treated with DAPT (10 mg/kg) in PBS (DAPT group), mice treated with PTE (100 mg/kg) and LY (20 mg/kg) in PBS (PTE+LY group) and mice treated with LY (20 mg/kg) in PBS (LY group). Mice were injected six times at 3-day intervals (on days 1, 4, 7, 10, 13 and 16). The tumor size was measured every 3 days using calipers (on days 2, 5, 8, 11, 14 and 17). On day 17, the tumors were excised from the euthanized mice for Western blot analysis.

### Western Blotting

The cell or tumor samples were lysed in sample buffer (150 mM Tris pH 6.8, 8 M urea, 50 mM DTT, 2% sodium dodecyl sulfate, 15% sucrose, 2 mM EDTA, 0.01% bromophenol blue, 1% protease and phosphatase inhibitor cocktails), sonicated, boiled, run through an 8–12% Bis/Tris gel using 5× MES buffer (Invitrogen) and transferred to an Immobilon NC membrane (Millipore). The membranes were blocked with 5% nonfat milk in TBST (150 mM NaCl, 50 mM Tris pH 7.5, 0.1% Tween-20) and then probed with antibodies against NICD, Hes1, Cyclin D1, survivin, Bax and Cytochrome c (1∶500) and antibodies against Nicastrin, Presenilin-1, p-Akt^ser473^, Akt, p-S6^Ser235/236^, S6, p-mTOR^ser2448^, mTOR, DNA-PK and GAPDH (1∶1000) overnight at 4°C. Then, the membranes were placed in blocking buffer, washed with TBST, probed with secondary antibodies (1∶5000) in blocking buffer at room temperature for 90 min and washed. The fluorescence was detected using a BioRad imaging system (BioRad, USA). The signals were quantified using Image Lab Software (BioRad, USA).

### Statistical Analyses

All of the experiments were performed in duplicate and repeated at least three times. The data are expressed as the mean ± the standard error of mean (SEM). The treatment groups were compared by a one-way variance (ANOVA) with SPSS 12.0 (SPSS Inc., Chicago) software. The differences were considered statistically significant at P<0.05.

## Results

### The Effects of PTE on the Apoptosis and Viability of Lung Adenocarcinoma Cells

**Figure 1:**
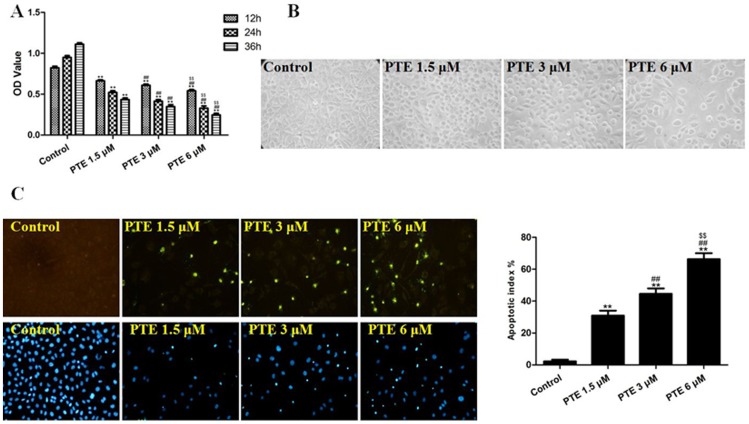
The effects of PTE on the viability, morphology and apoptotic index of lung adenocarcinoma cells. (A) Cells were treated with PTE at different concentrations (1.5, 3 and 6 µM) for different durations (12, 24 and 36 h). Cell viability was assessed using the MTT assay and was expressed as an OD value. (B) The cell morphology was observed under an inverted/phase contrast microscope (treated for 24 h), and images were taken (×200). Significant cell shrinkage and a decreased cellular attachment rate were observed in the PTE-treated group. (C) Apoptosis of the cells was detected by the TUNEL assay (×200), and the level of cell apoptosis was expressed as the apoptotic index. TUNEL staining was performed to stain the nuclei of apoptotic cells (green), and DAPI was used to stain the nuclei of all cells (blue). The apoptotic index was expressed as the number of green cells/the total number of cells counted×100%. The results are expressed as the mean ± SEM, n = 6. **P<0.01 compared with the control group, ^##^P<0.01 compared with the PTE 1.5 µM group, ^$$^P<0.01 compared with the PTE 3 µM group. PTE, pterostilbene; OD, optical density.

The viability of A549 cells treated with PTE was determined using an MTT assay, and the data are presented in Figure 1A. The treatment of A549 cells for 12, 24 or 36 h with 1.5, 3 or 6 µM of PTE resulted in cell growth inhibition in a dose- and time-dependent manner. The IC_50_ (50% inhibitory concentration) of PTE at 24 h was approximately 3.476 µM. The microscopy images (Figure 1B) showed that PTE treatment resulted in significant cell shrinkage and a decrease in the rate of cellular attachment compared with the control treatment. After treatment with 1.5, 3 or 6 µM of PTE for 24 h, the apoptotic index was increased in a dose-dependent manner (P<0.01, compared with the control group). These results provide convincing data showing that PTE can induce apoptosis of A549 cells (Figure 1C).

### The Effects of PTE on the Mitochondrial Membrane Potential in Lung Adenocarcinoma Cells

**Figure 2:**
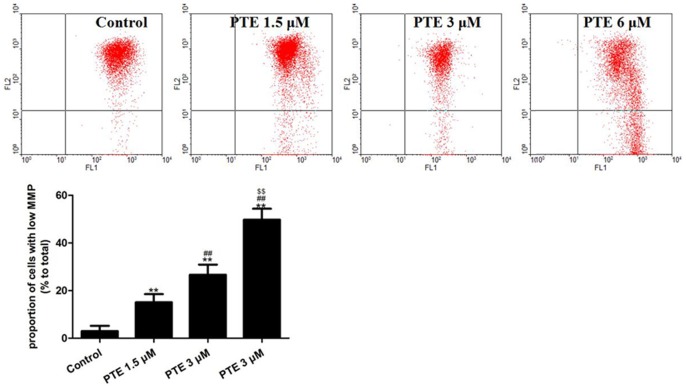
The effects of PTE on the MMP of lung adenocarcinoma cells treated for 24 h. The MMP was estimated by flow cytometry after staining the cells with the fluorescent dye JC-1. Representative flow cytometry results are shown. The subpopulations and their fractions are indicated: normal MMP cells (upper right) and low MMP cells (low right). The MMP level was expressed as the proportion of cells with a low MMP. The results are expressed as the mean ± SEM, n = 6. **P<0.01 compared with the control group, ^##^P<0.01 compared with the PTE 1.5 µM group, ^$$^P<0.01 compared with the PTE 3 µM group. PTE, pterostilbene; MMP, mitochondrial membrane potential.

After treatment with PTE (1.5, 3 or 6 µM) for 24 h, the proportion of cells with a low MMP increased significantly in a dose-dependent manner (P<0.01, compared with the control group, Figure 2). These results indicate that PTE can reduce the MMP of A549 cells.

### The Effects of PTE on ROS Generation and the GSH Level in Lung Adenocarcinoma Cells

**Figure 3:**
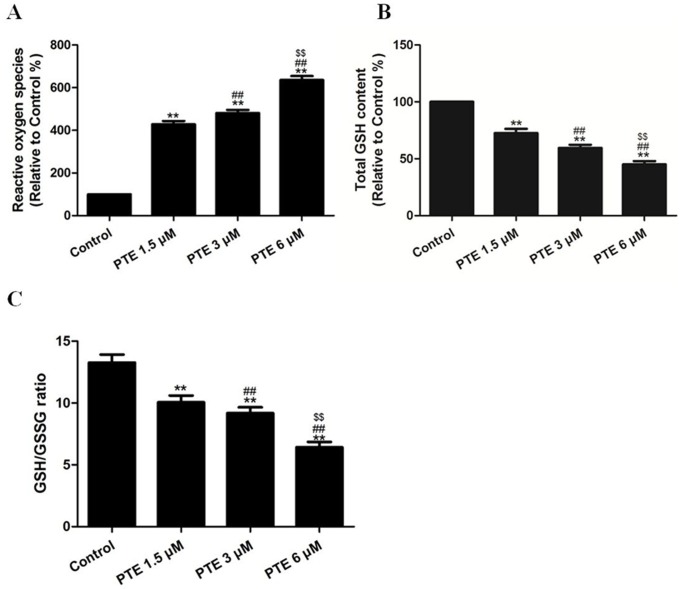
The effects of PTE on ROS generation, the GSH level and the GSH/GSSG ratio in lung adenocarcinoma cells treated for 24 h. (A) To determine the ROS concentration, the cells were loaded with DCFH-DA, and the fluorescence intensity was measured using a microplate fluorescence reader. The fluorescence intensity in the control group was defined as 100%. (B) The intracellular GSH content was measured using a GSH kit. The differences in the GSH level between groups were expressed as a percentage of the control. (C) The GSH and GSSG levels were evaluated by comparison with the standards and were normalized to the protein content. The results are expressed as the GSH/GSSG ratio. The results are expressed as the mean ± SEM, n = 6. **P<0.01 compared with the control group, ^##^P<0.01 compared with the PTE 1.5 µM group, ^$$^P<0.01 compared with the PTE 3 µM group. PTE, pterostilbene; ROS, reactive oxygen species; GSH, glutathione; GSSG, glutathione disulfide.

To measure the capacity of PTE to cause intracellular oxidation, the specific oxidation-sensitive fluorescent dye DCFH-DA was used. The fluorescent intensity of this dye is increased following the generation of intracellular reactive metabolites. The treatment of A549 cells with PTE (1.5, 3 or 6 µM) for 24 h induced a dose-dependent increase in ROS generation (P<0.01, compared with the control group, Figure 3A).

Reduced GSH is the major non-protein thiol in cells and is essential for maintaining the cellular redox status [21]. Because the PTE-induced apoptosis of A549 cells correlated with reactive oxygen species generation, we speculated that PTE treatment might disturb the cellular redox status. To address this possibility, the effect of PTE treatment on the intracellular GSH level was determined. The treatment of A549 cells with PTE (1.5, 3 or 6 µM) for 24 h induced a dose-dependent decrease in the intracellular GSH level (P<0.01, compared with the control group, Figure 3B). When GSH is converted into its oxidized form (GSSG), it must then be reduced by a combination of GSH reductase and NADPH. Therefore, one measure of the cellular oxidative status is the ratio of the levels of the reduced and oxidized forms of GSH. PTE induced a dose-dependent decrease in the GSH/GSSG ratio in A549 cells (Figure 3C). These results support the hypothesis that PTE treatment affects the cellular redox status of the cells.

### The Effects of PTE on Proteins in the Notch1 Signaling Pathway and the Mitochondrial Apoptotic Pathway in Lung Adenocarcinoma Cells

**Figure 4:**
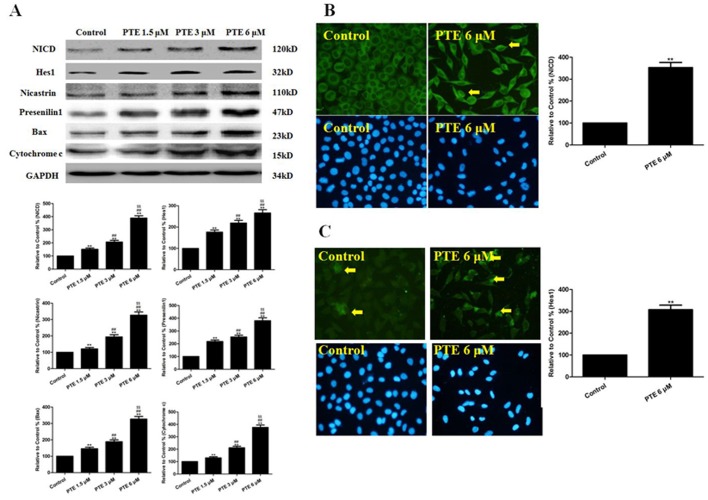
The effects of PTE on proteins in the Notch1 signaling pathway and the mitochondrial apoptotic pathway in lung adenocarcinoma cells treated for 24 h. (A) Representative Western blot results are shown. The PTE treatment increased the expression levels of NICD, Hes1, Nicastrin, Presenilin-1, Bax and Cytochrome c. (B) Representative images of Notch1 immunofluorescence are shown (×400). (C) Representative images of Hes1 immunofluorescence are shown (×400). The results are expressed as the mean ± SEM, n = 6. **P<0.01 compared with the control group, ^##^P<0.01 compared with the PTE 1.5 µM group, ^$$^P<0.01 compared with the PTE 3 µM group. PTE, pterostilbene.

The Western blot and immunofluorescence results showed that PTE increased the NICD protein level in A549 cells (Figure 4A and 4B). We then determined if the increase in the NICD protein level could augment Notch1 signaling by measuring the activity of a downstream target, Hes1. As observed for the NICD protein, PTE induced Hes1 expression (Figure 4A and 4C). We then examined the possible mechanism underlying the induction of NICD expression by PTE treatment. NICD is produced when the Notch1 receptor is cleaved by the gamma secretase complex, which is composed of four subunits (Presenilin-1, Nicastrin, anterior pharynx-defective 1 (Aph-1) and presenilin enhancer 2 (Pen-2)) [11], [12]. Therefore, PTE treatment induced NICD protein expression at least partially by increasing the gamma secretase activity. The treatment of A549 cells with PTE induced the expression of two of the subunits, Presenilin-1 and Nicastrin (Figure 4A). In addition, the expression levels proteins related to the mitochondrial apoptotic pathway (Bax and Cytochrome c) were up-regulated by PTE treatment, indicating that this apoptotic pathway was activated (Figure 4A).

### The Effects of PTE Combined with DAPT or Notch1 siRNA on the Viability, Apoptotic Index and Notch1 Signaling of Lung Adenocarcinoma Cells

**Figure 5:**
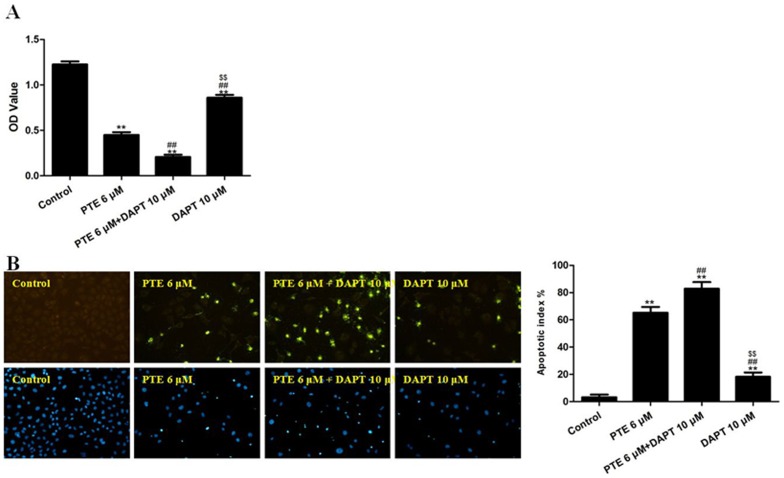
The effects of PTE combined with DAPT on the viability and apoptotic index of lung adenocarcinoma cells treated for 24 h. (A) Cell viability was assessed using the MTT assay, and the viability was expressed as an OD value. (B) Apoptosis was detected by the TUNEL assay (×200) and was expressed as the apoptotic index. TUNEL staining was performed to stain the nuclei of the apoptotic cells (green), and DAPI was used to stain the nuclei of all cells (blue). The apoptotic index was expressed as the number of green cells/the total number of cells counted×100%. The results are expressed as the mean ± SEM, n = 6. **P<0.01 compared with the control group, ^##^P<0.01 compared with the PTE 6 µM group, ^$$^P<0.01 compared with the PTE 6 µM+DAPT 10 µM group. PTE, pterostilbene; OD, optical density.

**Figure 6:**
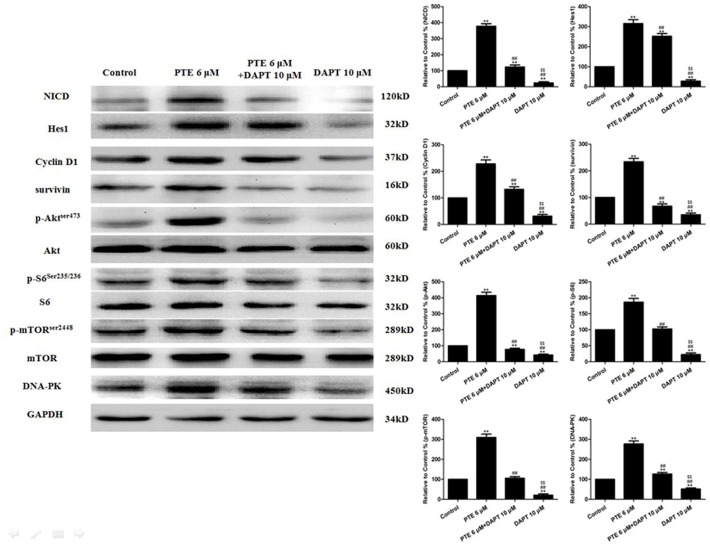
The effects of PTE combined with DAPT on the Notch1 signaling and pro-survival factors in lung adenocarcinoma cells treated for 24 h. Representative Western blot results are shown. The results are expressed as the mean ± SEM, n = 6. **P<0.01 compared with the control group, ^##^P<0.01 compared with the PTE 6 µM group, ^$$^P<0.01 compared with the PTE 6 µM+DAPT 10 µM group. PTE, pterostilbene.

A549 cells were treated with PTE (6 µM) combined with DAPT for 24 h, and the cell viability was then determined using an MTT assay. Treatment with PTE or DAPT alone decreased the cell viability (P<0.01, compared with the control group, Figure 5A). The combination of DAPT and PTE further decreased the cell viability (P<0.01, compared with the PTE 6 µM group or the DAPT 10 µM group, Figure 5A). In addition, the co-treatment of A549 cells with DAPT and PTE significantly increased the percentage of apoptotic cells compared with either treatment alone (P<0.01, Figure 5B). As expected, treatment with PTE and DAPT further inhibited NICD and Hes1 expression (Figure 6).

**Figure 7:**
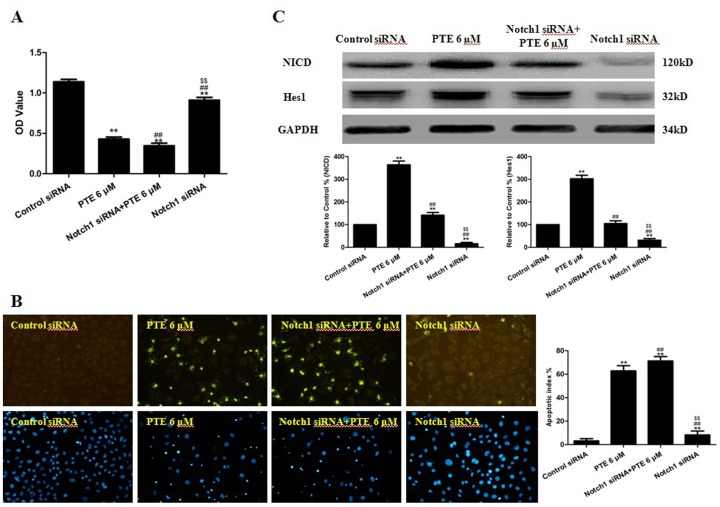
The effects of PTE combined with Notch1 siRNA on the viability, apoptotic index and Notch1 signaling of lung adenocarcinoma cells. (A) Cell viability was assessed using the MTT assay and was expressed as an OD value. (B) Apoptosis was detected by the TUNEL assay (×200) and was expressed as the apoptotic index. TUNEL staining was performed to stain the nuclei of the apoptotic cells (green), and DAPI was used to stain the nuclei of all cells (blue). The apoptotic index was expressed as the number of green cells/the total number of cells counted×100%. (C) Representative Western blot results are shown. The results are expressed as the mean ± SEM, n = 6. **P<0.01 compared with the control siRNA group, ^##^P<0.01 compared with the PTE 6 µM group, ^$$^P<0.01 compared with the Notch siRNA PTE 6 µM group. PTE, pterostilbene; OD, optical density.

Because the gamma secretase complex cleaves multiple transmembrane receptors other than Notch1 (including the other Notch receptors, Notch2, 3 and 4), it was necessary to confirm that the sensitization effect of DAPT was mediated by Notch1. We used Notch1 siRNA (to specifically inhibit Notch1) to determine if the sensitization effect observed for DAPT could be replicated. We transfected the cells with Notch1 siRNA for 48 h to decrease expression of NICD (Figure 7C), and this treatment also decreased the expression of Hes1 (Figure 7C). Compared with the cells in the control group, the A549 cells in the Notch1 siRNA-treated group had a decreased viability (P<0.01, compared with the control group, Figure 7A). Similar to the results obtained with DAPT, the combination of Notch1 siRNA and PTE decreased the cell viability significantly (P<0.01, compared with the PTE 6 µM group or the Notch1 siRNA group, Figure 7A). In addition, the co-treatment of A549 cells with Notch1 siRNA and PTE significantly increased the percentage of apoptotic cells (P<0.01, compared with the PTE 6 µM group or the Notch1 siRNA group, Figure 7B).

### The Effects of DAPT on the Induction of Pro-survival Factors, Including Akt Signaling Proteins, by PTE

**Figure 8:**
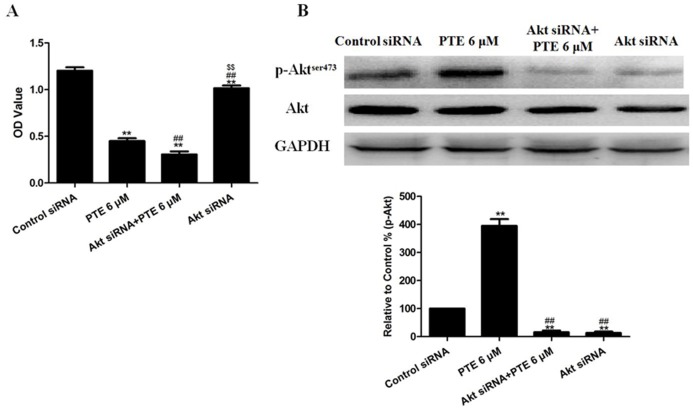
The effects of PTE combined with Akt siRNA on the viability and Akt signaling of lung adenocarcinoma cells. (A) Cell viability was assessed using the MTT assay and was expressed as an OD value. (B) Representative Western blot results are shown. The results are expressed as the mean ± SEM, n = 6. **P<0.01 compared with the control siRNA group, ^##^P<0.01 compared with the PTE 6 µM group, ^$$^P<0.01 compared with the Akt siRNA+PTE 6 µM group. PTE, pterostilbene; OD, optical density.

The treatment of A549 cells with DAPT decreased the baseline levels of Cyclin D1 and survivin and suppressed the induction of Cyclin D1 and survivin by PTE (Figure 6). Because Notch1 may activate PI3K/Akt signaling to protect against DNA damage [17], the expression levels of proteins in this pathway were examined. In A549 cells, co-treatment with DAPT decreased the phosphorylation of Akt at Serine 473 induced by PTE and also mildly decreased the total Akt level (Figure 6). We then examined two targets of Akt, mTOR and the S6 ribosomal protein. The levels of S6 phosphorylated at Serine 235/236, mTOR phosphorylated at Serine 2448, total S6 and total mTOR were decreased following co-treatment with DAPT and PTE (Figure 6). Then, the expression of DNA-PK, a kinase that may activate Akt, was analyzed. We found that co-treatment with DAPT and PTE decreased the protein level of DNA-PK (Figure 6). Because functional Akt signaling may play a role in chemoresistance, we determined if the suppression of Akt with siRNA would alter the sensitization effect of PTE. Akt siRNA not only effectively reduced the levels of phosphorylated and total Akt protein (Figure 8B) but also decreased the viability (Figure 8A) of A549 cells following PTE treatment.

### The Effects of PTE Combined with DAPT or LY on Tumor Xenografts in vivo

**Figure 9:**
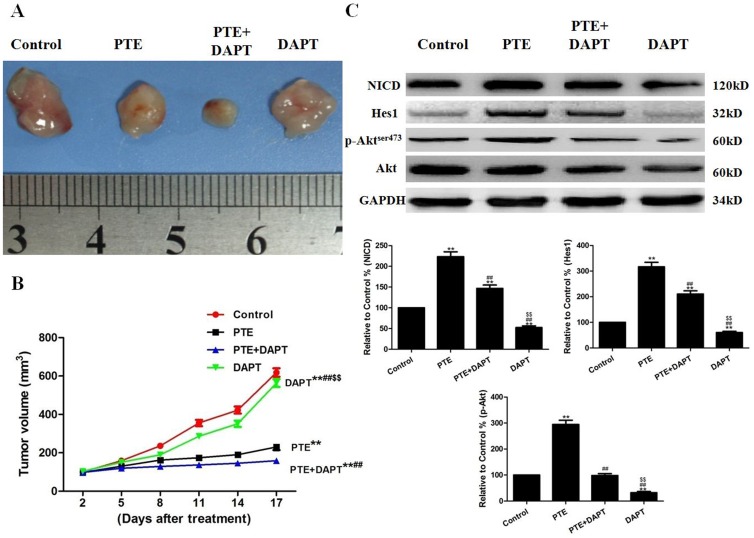
The effects of PTE combined with DAPT on tumor xenografts in vivo. (A) Photographs showing the morphology of the tumor xenografts in different groups. (B) The tumor growth curve was drawn using the tumor volume and the treatment duration. (C) Representative Western blot results are shown. The results are expressed as the mean ± SEM, n = 3. **P<0.01 compared with the control group, ^##^P<0.01 compared with the PTE group, ^$$^P<0.01 compared with the PTE+DAPT group. PTE, pterostilbene.

**Figure 10:**
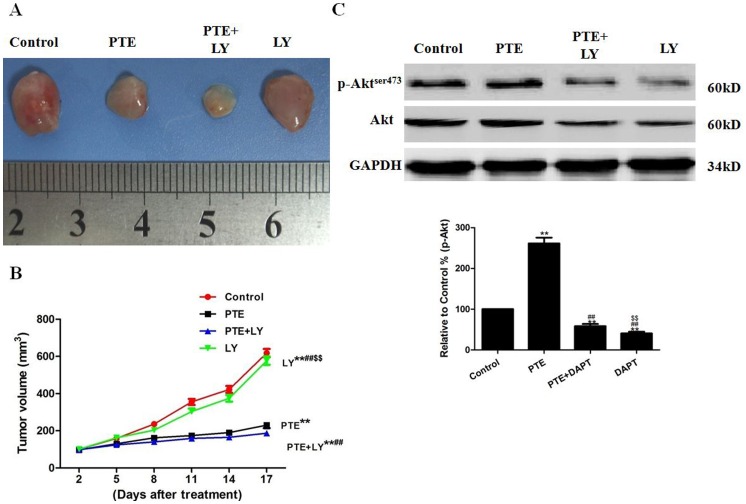
The effects of PTE combined with LY on tumor xenografts in vivo. (A) Photographs showing the morphology of the tumor xenografts in different groups. (B) The tumor growth curve was drawn using the tumor volume and the treatment duration. (C) Representative Western blot results are shown. The results are expressed as the mean ± SEM, n = 3. **P<0.01 compared with the control group, ^##^P<0.01 compared with the PTE group, ^$$^P<0.01 compared with the PTE+LY group. PTE, pterostilbene; LY, LY294002.

To determine whether PTE can inhibit tumor growth in animals, we established A549 xenografts in athymic nude mice. We found that the mice in all treatment groups developed subcutaneous tumors. As shown in Figures 9A and 9B, treatment with PTE or DAPT alone significantly inhibited tumor growth (P<0.01, compared with the control group). The combination of DAPT and PTE further inhibited tumor growth (P<0.01, compared with the PTE group or the DAPT group). To further investigate whether PTE or/and DAPT regulates Notch1 signaling in vivo, we examined NICD and Hes1 expression in tumor tissues. Western blot analysis showed that the expression levels of NICD and Hes1 were significantly higher in tumors from the PTE-treated mice (P<0.01, compared with the control group, Figure 9C). As expected, co-treatment with PTE and DAPT inhibited NICD and Hes1 expression (P<0.01, compared with the PTE group, Figure 9C). In addition, co-treatment with PTE and DAPT decreased the phosphorylation of Akt at Serine 473 (P<0.01, compared with the PTE group). Next, we determined if the suppression of Akt with LY would alter the sensitization effect of PTE. LY not only effectively suppressed the level of phosphorylated Akt protein (Figure 10C) but also further enhanced the tumor growth inhibition induced by PTE treatment (Figures 10A and 10B).

## Discussion

As a natural dimethylated analog of resveratrol, PTE has been shown to suppress the proliferation of various types of cancer cells, including pancreatic, breast, colon, oral, lung and prostate carcinoma cells, as well as melanoma, myeloma and leukemia cells [5]–[7]. Various molecules and signaling pathways are involved in the anti-tumor effects of PTE, including cytosolic Ca^2+^ overload [5], adenosine monophosphate activated protein kinase (AMPK) signaling [7], autophagy [22], ROS [22], Wnt signaling [23] and lysosomal membrane permeabilization [24]. Studies have shown that resveratrol suppresses the tumorigenicity of breast cancer, glioblastoma and medullary thyroid cancer by regulating the Notch axis [9], [10], [25]. However, the effects of PTE of human lung adenocarcinoma and the mechanisms responsible for these effects are not fully understood. In our study, PTE treatment resulted in a dose- and time-dependent inhibition of the viability of lung adenocarcinoma cells. In addition, PTE treatment also significantly inhibited tumor growth in A549 xenografts in athymic nude mice.

GSH is the main non-protein antioxidant in the cell. GSH can inactivate superoxide anion free radicals and provide electrons for enzymes such as glutathione peroxidase, which reduces H_2_O_2_ to H_2_O. Reduced GSH is the major non-protein thiol in cells and is essential for maintaining the cellular redox status. The intracellular GSH content has a decisive effect on anticancer drug-induced apoptosis, and the apoptotic effects are inversely proportional to the GSH content [21]. The MMP is produced when protons are pumped from the mitochondrial matrix into the inter-membrane space. Decreases in the MMP have been linked to apoptotic pathways, including the mitochondrial apoptotic pathway [26]. During the process of apoptosis, mitochondria are a source of ROS generated by the reduced MMP, and the enhancement of ROS production has long been associated with the apoptotic response induced by anticancer agents [27]. Our results show that PTE can significantly decrease the MMP and increase ROS production in lung adenocarcinoma cells. Likewise, our results clearly indicate there the intracellular GSH content was depleted by PTE treatment. Importantly, PTE treatment also up-regulated the expression of the mitochondrial apoptotic pathway-related proteins Bax and Cytochrome c.

Studies have shown that components of the Notch1 pathway are overexpressed during the progression of lung adenocarcinoma, as observed for other genes involved in the survival of cancer cells [13], [28]. The protein levels of downstream targets are also elevated, and the overexpression of Hes1 has been previously reported in lung adenocarcinoma [13], [29]. Gamma secretase is a multi-protein complex containing an intra-membrane cleaving protease. This complex has a growing list of protein substrates, including the Notch receptors. The four components of the gamma secretase complex (Presenilin-1, Nicastrin, Pen-2 and Aph-1) are all thought to be essential for its activity. The catalytic domain is located within Presenilin-1, and Nicastrin has been suggested to be critical for substrate recognition [12]. Our results indicate that PTE treatment induced the NICD protein and activated Hes1 in vitro and in vivo. PTE treatment also resulted in the up-regulation of Presenilin-1 and Nicastrin. These data suggest that PTE induces the activation of the Notch1 signaling pathway in part through the activation of the gamma secretase complex.

Because the activation of Notch1 signaling contributes to the survival of cancer cells, the inhibition of the Notch1 pathway can sensitize cells to chemotherapy [30]. Our results indicate that the viability of lung adenocarcinoma cells in vitro and the growth of tumor xenografts in vivo were both further decreased by the use of DAPT in combination with PTE treatment. These decreases were associated with the inhibition of NICD production and the suppression of Hes1 activity. Our results indicate that the use of DAPT may be a novel means to both enhance the effects of chemotherapy and delay chemoresistance in patients with cancer [31]. However, this effect of DAPT may be tumor specific. For example, in neuroendocrine cells, Notch1 may act as a tumor suppressor [32]. Therefore, it remains to be determined whether this effect of DAPT on the effects of chemotherapy can be extended to all tumor sub-types. Because DAPT also prevents the cleavage of other transmembrane proteins, we performed several siRNA experiments. Our studies indicate that in lung adenocarcinoma cancer cells, the selective suppression of Notch1 with siRNA enhances the effects of PTE treatment. Interestingly, the degree of sensitization to PTE mediated by Notch1 siRNA was not as significant as that observed after treatment with DAPT. Because DAPT targets all four of the Notch receptors, we will perform further experiments in which siRNA is used to knock down the expression of each Notch receptor to determine their individual effects on PTE treatment.

The possible mechanisms by which DAPT enhances the effect of PTE treatment in lung adenocarcinoma cells appear to be multifactorial. Previous studies have shown that DAPT can suppress the expression Cyclin D1 and survivin, two pro-survival factors that are targets of Notch1 and have been reported to play a role in sensitization to anticancer drugs [33]–[35]. Notch1 signaling has also been implicated in Akt activation. For example, it is known that Notch1 can activate Akt in cervical cancer [36], glioma [37] and leukemia [38]. In addition, a recent microarray study identified the overactivation of the Akt and Notch1 signaling pathways as hallmarks of poor prognosis for glioma patients [39]. Other studies have also reported a link between the Akt/mTOR pathways and Notch1. For example, the protective effect of NICD against p53-mediated apoptosis is abrogated when mTOR is inhibited [14]. In lymphoid cell lines, co-transfection with a dominant-negative Akt mutant decreased the Notch1-mediated protection against apoptosis [17]. Therefore, we investigated the mechanisms by which the inhibition of Notch1 signaling may enhance PTE treatment. First, we determined if DAPT co-treatment could decrease the expression several pro-survival factors implicated in cancer survival pathways. The treatment of lung adenocarcinoma cells with DAPT decreased the baseline level of survivin and suppressed the induction of Cyclin D1 by PTE. Second, because Notch1 may activate PI3K/Akt signaling to protect against DNA damage [12], the expression levels of proteins in this pathway were examined. In lung adenocarcinoma cells and tumor xenografts, co-treatment with DAPT decreased the phosphorylation of Akt at Serine 473 induced by PTE and also mildly decreased the total Akt level. We then examined two targets of Akt, mTOR and the S6 ribosomal protein, in lung adenocarcinoma cells. The levels of S6 phosphorylated at Serine 235/236, mTOR phosphorylated at Serine 2448, total S6 and total mTOR were all decreased following co-treatment with DAPT and PTE. We then examined the expression of a kinase that may activate Akt (DNA-PK), and we found that co-treatment with DAPT and PTE decreased the protein level of DNA-PK. Intact Akt signaling may play a role in cancer survival pathways, and thus, we determined if the suppression of Akt with siRNA in vitro or with LY in vivo would affect the anticancer activity of PTE. Our results confirmed that Akt siRNA and LY effectively reduced the total Akt protein level and decreased the viability of lung adenocarcinoma cells and the growth of tumor xenografts following PTE treatment. These results suggest that the inhibition of Notch1 signaling may sensitize lung adenocarcinoma cells to PTE by preventing the activation of pro-survival pathways, including the Akt pathway, after DNA damage.

In conclusion, these experiments provide mechanistic evidence that the Notch1 pathway is activated in lung adenocarcinoma cells in response to PTE treatment to enhance the survival of cancer cells. The down-regulation of Notch1 signaling sensitizes lung adenocarcinoma cells to PTE treatment. Additionally, this potentiation of chemosensitivity by Notch inhibition may be related to the down-regulation of pro-survival pathways. Therefore, we propose that the inhibition of Notch1 signaling may be a novel strategy to prevent the induction of cancer survival mechanisms in advanced lung adenocarcinomas.

## References

[1] Ren XL, Xu YM, Bao W, Fu HJ, Wu CG, et al (2009) Inhibition of non-small cell lung cancer cell proliferation and tumor growth by vector-based small interfering RNAs targeting HER2/neu. Cancer Lett 281: 134–143. 19339104 10.1016/j.canlet.2009.02.036

[2] Jemal A, Siegel R, Ward E, Hao Y, Xu J, et al (2008) Cancer statistics, 2008. CA Cancer J Clin 58: 71–96. 18287387 10.3322/CA.2007.0010

[3] McCormack D, McFadden D (2012) Pterostilbene and cancer: current review. J Surg Res 173: e53–61. 22099605 10.1016/j.jss.2011.09.054

[4] Ruiz MJ, Fernández M, Picó Y, Mañes J, Asensi M, et al (2009) Dietary administration of high doses of pterostilbene and quercetin to mice is not toxic. J Agric Food Chem 57: 3180–3186. 19292443 10.1021/jf803579e

[5] Moon D, McCormack D, McDonald D, McFadden D (2012) Pterostilbene induces mitochondrially derived apoptosis in breast cancer cells in vitro. J Surg Res 180: 208–215. 22572619 10.1016/j.jss.2012.04.027

[6] Pan MH, Chiou YS, Chen WJ, Wang JM, Badmaev V, et al (2009) Pterostilbene inhibited tumor invasion via suppressing multiple signal transduction pathways in human hepatocellular carcinoma cells. Carcinogenesis 30: 1234–1242. 19447859 10.1093/carcin/bgp121

[7] Lin VC, Tsai YC, Lin JN, Fan LL, Pan MH, et al (2012) Activation of AMPK by pterostilbene suppresses lipogenesis and cell-cycle progression in p53 positive and negative human prostate cancer cells. J Agric Food Chem 60: 6399–6407. 22670709 10.1021/jf301499e

[8] Cichocki M, Paluszczak J, Szaefer H, Piechowiak A, Rimando AM, et al (2008) Pterostilbene is equally potent as resveratrol in inhibiting 12-O-tetradecanoylphorbol-13-acetate activated NFkappaB, AP-1, COX-2, and iNOS in mouse epidermis. Mol Nutr Food Res 52: S62–S70. 18551458 10.1002/mnfr.200700466

[9] Truong M, Cook MR, Pinchot SN, Kunnimalaiyaan M, Chen H (2011) Resveratrol induces Notch2-mediated apoptosis and suppression of neuroendocrine markers in medullary thyroid cancer. Ann Surg Oncol 18: 1506–1511. 21184191 10.1245/s10434-010-1488-z PMC3078954

[10] Lin H, Xiong W, Zhang X, Liu B, Zhang W, et al (2011) Notch-1 activation-dependent p53 restoration contributes to resveratrol-induced apoptosis in glioblastoma cells. Oncol Rep 26: 925–930. 21743969 10.3892/or.2011.1380

[11] Androutsellis-Theotokis A, Leker RR, Soldner F, Hoeppner DJ, Ravin R, et al (2006) Notch signalling regulates stem cell numbers in vitro and in vivo. Nature 442: 823–826. 16799564 10.1038/nature04940

[12] Meng RD, Shelton CC, Li YM, Qin LX, Notterman D, et al (2009) gamma-Secretase inhibitors abrogate oxaliplatin-induced activation of the Notch-1 signaling pathway in colon cancer cells resulting in enhanced chemosensitivity. Cancer Res 69: 573–582. 19147571 10.1158/0008-5472.CAN-08-2088 PMC3242515

[13] Allen TD, Rodriguez EM, Jones KD, Bishop JM (2011) Activated Notch1 induces lung adenomas in mice and cooperates with Myc in the generation of lung adenocarcinoma. Cancer Res 71: 6010–6018. 21803744 10.1158/0008-5472.CAN-11-0595 PMC3174331

[14] Mungamuri SK, Yang X, Thor AD, Somasundaram K (2006) Survival signaling by Notch1: mammalian target of rapamycin (mTOR)-dependent inhibition of p53. Cancer Res 66: 4715–4724. 16651424 10.1158/0008-5472.CAN-05-3830

[15] Stylianou S, Clarke RB, Brennan K (2006) Aberrant activation of notch signaling in human breast cancer. Cancer Res 66: 1517–1525. 16452208 10.1158/0008-5472.CAN-05-3054

[16] Nair P, Somasundaram K, Krishna S (2003) Activated Notch1 inhibits p53-induced apoptosis and sustains transformation by human papillomavirus type 16 E6 and E7 oncogenes through a PI3K-PKB/Akt-dependent pathway. J Virol 77: 7106–7112. 12768030 10.1128/JVI.77.12.7106-7112.2003 PMC156194

[17] Sade H, Krishna S, Sarin A (2004) The anti-apoptotic effect of Notch-1 requires p56lck-dependent, Akt/PKB-mediated signaling in T cells. J Biol Chem 279: 2937–2944. 14583609 10.1074/jbc.M309924200

[18] Chen Y, Li D, Liu H, Xu H, Zheng H, et al (2011) Notch-1 signaling facilitates survivin expression in human non-small cell lung cancer cells. Cancer Biol Ther 11: 14–21. 20962575 10.4161/cbt.11.1.13730

[19] Ji X, Wang Z, Geamanu A, Sarkar FH, Gupta SV (2011) Inhibition of cell growth and induction of apoptosis in non-small cell lung cancer cells by delta-tocotrienol is associated with notch-1 down-regulation. J Cell Biochem 112: 2773–2783. 21598300 10.1002/jcb.23184

[20] Huang PH, Chen JS, Tsai HY, Chen YH, Lin FY, et al (2011) Globular adiponectin improves high glucose-suppressed endothelial progenitor cell function through endothelial nitric oxide synthase dependent mechanisms. J Mol Cell Cardiol 51: 109–119. 21439968 10.1016/j.yjmcc.2011.03.008

[21] Zhao YF, Zhang C, Suo YR (2012) MMPT as a reactive oxygen species generator induces apoptosis via the depletion of intracellular GSH contents in A549 cells. Eur J Pharmacol 688: 6–13. 22609960 10.1016/j.ejphar.2012.05.003

[22] Chakraborty A, Bodipati N, Demonacos MK, Peddinti R, Ghosh K, et al (2012) Long term induction by pterostilbene results in autophagy and cellular differentiation in MCF-7 cells via ROS dependent pathway. Mol Cell Endocrinol 355: 25–40. 22273805 10.1016/j.mce.2012.01.009

[23] Zhang W, Sviripa V, Kril LM, Chen X, Yu T, et al (2011) N-dialkylaminostilbenes for Wnt pathway inhibition and colon cancer repression. J Med Chem 54: 1288–1297. 21291235 10.1021/jm101248v PMC3073490

[24] Mena S, Rodríguez ML, Ponsoda X, Estrela JM, Jäättela M, et al (2012) Pterostilbene-induced tumor cytotoxicity: a lysosomal membrane permeabilization-dependent mechanism. PLoS One 7: e44524. 22957077 10.1371/journal.pone.0044524 PMC3434130

[25] Li Y, Wicha MS, Schwartz SJ, Sun D (2011) Implications of cancer stem cell theory for cancer chemoprevention by natural dietary compounds. J Nutr Biochem 22(9): 799–806. 21295962 10.1016/j.jnutbio.2010.11.001 PMC3248810

[26] Shen XJ, Wang HB, Ma XQ, Chen JH (2012) β,β-Dimethylacrylshikonin Induces Mitochondria Dependent Apoptosis through ERK Pathway in Human Gastric Cancer SGC-7901 Cells. PLoS One 7: e41773. 22848597 10.1371/journal.pone.0041773 PMC3407073

[27] Liu Z, Li D, Zhao W, Zheng X, Wang J, et al (2012) A potent lead induces apoptosis in pancreatic cancer cells. PLoS One 7: e37841. 22745658 10.1371/journal.pone.0037841 PMC3380052

[28] Westhoff B, Colaluca IN, D'Ario G, Donzelli M, Tosoni D, et al (2009) Alterations of the Notch pathway in lung cancer. Proc Natl Acad Sci U S A 106: 22293–22298. 20007775 10.1073/pnas.0907781106 PMC2799768

[29] Somasundaram K, Reddy SP, Vinnakota K, Britto R, Subbarayan M, et al (2005) Upregulation of ASCL1 and inhibition of Notch signaling pathway characterize progressive astrocytoma. Oncogene 24: 7073–7083. 16103883 10.1038/sj.onc.1208865

[30] McAuliffe SM, Morgan SL, Wyant GA, Tran LT, Muto KW, et al (2012) Targeting Notch, a key pathway for ovarian cancer stem cells, sensitizes tumors to platinum therapy. Proc Natl Acad Sci U S A 109: E2939–2948. 23019585 10.1073/pnas.1206400109 PMC3491453

[31] Akiyoshi T, Nakamura M, Yanai K, Nagai S, Wada J, et al (2008) Gamma-secretase inhibitors enhance taxane-induced mitotic arrest and apoptosis in colon cancer cells. Gastroenterology 134: 131–144. 18166351 10.1053/j.gastro.2007.10.008

[32] Wang H, Chen Y, Fernandez-Del Castillo C, Yilmaz O, Deshpande V (2013) Heterogeneity in signaling pathways of gastroenteropancreatic neuroendocrine tumors: a critical look at notch signaling pathway. Mod Pathol 26: 139–147. 22918166 10.1038/modpathol.2012.143

[33] Chen Y, De Marco MA, Graziani I, Gazdar AF, Strack PR, et al (2007) Oxygen concentration determines the biological effects of NOTCH-1 signaling in adenocarcinoma of the lung. Cancer Res 67: 7954–7959. 17804701 10.1158/0008-5472.CAN-07-1229

[34] Naganuma S, Whelan KA, Natsuizaka M, Kagawa S, Kinugasa H, et al (2012) Notch receptor inhibition reveals the importance of cyclin D1 and Wnt signaling in invasive esophageal squamous cell carcinoma. Am J Cancer Res 2: 459–475. 22860235 PMC3410579

[35] Chen Y, Li D, Liu H, Xu H, Zheng H, et al (2011) Notch-1 signaling facilitates survivin expression in human non-small cell lung cancer cells. Cancer Biol Ther 11: 14–21. 20962575 10.4161/cbt.11.1.13730

[36] Franko-Tobin LG, Mackey LV, Huang W, Song X, Jin B, et al (2012) Notch1-mediated tumor suppression in cervical cancer with the involvement of SST signaling and its application in enhanced SSTR-targeted therapeutics. Oncologist 17: 220–232. 22291092 10.1634/theoncologist.2011-0269 PMC3286171

[37] Muto J, Imai T, Ogawa D, Nishimoto Y, Okada Y, et al (2012) RNA-binding protein Musashi1 modulates glioma cell growth through the post-transcriptional regulation of Notch and PI3 kinase/Akt signaling pathways. PLoS One 7: e33431. 22428049 10.1371/journal.pone.0033431 PMC3299785

[38] Guo D, Teng Q, Ji C (2011) NOTCH and phosphatidylinositide 3-kinase/phosphatase and tensin homolog deleted on chromosome ten/AKT/mammalian target of rapamycin (mTOR) signaling in T-cell development and T-cell acute lymphoblastic leukemia. Leuk Lymphoma 52: 1200–1210. 21463127 10.3109/10428194.2011.564696

[39] Chan SM, Weng AP, Tibshirani R, Aster JC, Utz PJ (2007) Notch signals positively regulate activity of the mTOR pathway in T-cell acute lymphoblastic leukemia. Blood 110: 278–286. 17363738 10.1182/blood-2006-08-039883 PMC1896117

